# The effect of cyclic stretch on aortic viscoelasticity and the putative role of smooth muscle focal adhesion

**DOI:** 10.3389/fphys.2023.1218924

**Published:** 2023-08-11

**Authors:** Cédric H. G. Neutel, Callan D. Wesley, Guido R. Y. De Meyer, Wim Martinet, Pieter-Jan Guns

**Affiliations:** Laboratory of Physiopharmacology, University of Antwerp, Campus Drie Eiken, Antwerp, Belgium

**Keywords:** viscoelasticity, aorta, vascular smooth muscle cell, focal adhesion, cyclic stretch

## Abstract

Due to its viscoelastic properties, the aorta aids in dampening blood pressure pulsatility. At the level of resistance-arteries, the pulsatile flow will be transformed into a continuous flow to allow for optimal perfusion of end organs such as the kidneys and the brain. In this study, we investigated the *ex vivo* viscoelastic properties of different regions of the aorta of healthy C57Bl6/J adult mice as well as the interplay between (altered) cyclic stretch and viscoelasticity. We demonstrated that the viscoelastic parameters increase along the distal aorta and that the effect of altered cyclic stretch is region dependent. Increased cyclic stretch, either by increased pulse pressure or pulse frequency, resulted in decreased aortic viscoelasticity. Furthermore, we identified that the vascular smooth muscle cell (VSMC) is an important modulator of viscoelasticity, as we have shown that VSMC contraction increases viscoelastic parameters by, in part, increasing elastin fiber tortuosity. Interestingly, an acute increase in stretch amplitude reverted the changes in viscoelastic properties induced by VSMC contraction, such as a decreasing contraction-induced elastin fiber tortuosity. Finally, the effects of altered cyclic stretch and VSMC contraction on viscoelasticity were more pronounced in the abdominal infrarenal aorta, compared to both the thoracic ascending and descending aorta, and were attributed to the activity and stability of VSMC focal adhesion. Our results indicate that cyclic stretch is a modulator of aortic viscoelasticity, acting on VSMC focal adhesion. Conditions of (acute) changes in cyclic stretch amplitude and/or frequency, such as physical exercise or hypertension, can alter the viscoelastic properties of the aorta.

## 1 Introduction

The aorta is continuously exposed to cyclic deformation due to the pulsatile nature of blood pressure. Central arteries, such as the thoracic ascending and descending aorta, are subjected to high pulsatile stretching and act as cushions for the highly energetic cardiac output. This cushioning function (i.e., Windkessel effect) is an important feature, since it aids in dampening the pulsatile character of blood pressure and, at the level of the resistance arteries, assures a continuous blood flow. The importance of their damping function becomes clear when the aorta stiffens, for instance during arterial ageing (De Moudt et al., 2022). Central artery stiffening increases the transmission of pulsatile pressure to the periphery, which damages end-organs such as the brain and the kidneys (Mitchell, 2018; Chirinos et al., 2019; Aghilinejad et al., 2020). Measuring arterial stiffness is therefore an interesting and valuable method in predicting cardiovascular risk (Mitchell et al., 2010; Boutouyrie et al., 2021). There are different *in vivo* and *ex vivo* methods for measuring arterial stiffness, such as measuring pulse wave velocity in patients or analyzing arterial biomechanics in dedicated *ex vivo* setups, and they have been extensively reviewed elsewhere (Butlin et al., 2020; Segers et al., 2020). In general, unravelling the (altered) material properties of aortic tissue in disease provides an understanding of the pathophysiological mechanisms that are at play.

Importantly, the aorta exhibits both viscous and elastic behavior that contribute to dissipating and storing pulsatile energy, respectively. The viscoelastic behavior of the aorta manifests itself as the occurrence of hysteresis in stress-strain or pressure-diameter relationships (Remington, 1955; Bergel, 1961; Stefanadis et al., 1995). This means that the course of the loading phase (i.e., dilation) and unloading phase (i.e., recoil) are not equal. The importance of the viscous component in a viscoelastic conduit structure and its effect on hemodynamics were demonstrated experimentally in a study by Elliot et al. (Elliott et al., 2020). The authors demonstrated that the viscous properties of a viscoelastic tube dampen both up- and downstream blood flow pulsatility while also attenuating both wall shear stress and overall mechanical energy of flow (Elliott et al., 2020). Therefore, changes in the viscous properties of blood vessels, as seen as in hypertension and aortic aneurysms, will unfavorably affect hemodynamics and ventricular afterload, creating a worsening vicious circle in developing cardiovascular diseases (Armentano et al., 1995; Wang et al., 2013; Chung et al., 2014). Both the extracellular matrix and residing vascular cells, as well as the complex interplay between them, have been investigated as modulators of blood vessel viscoelasticity.

Vascular cells are tethered to both the extracellular matrix and adjacent vascular cells through adhesion receptors such as integrins and cadherins (Moiseeva, 2001). Integrins, for example, connect matrix components such as collagens to the actin cytoskeleton, mediating cell-matrix mechanotransduction (Goldschmidt et al., 2001; Delon and Brown, 2007). Moreover, the contractile state of the vascular smooth muscle cell (VSMC) has been shown to be coupled with its adhesion to the extracellular matrix, with constriction (and relaxation) leading to increased (and decreased) adhesion probability, respectively (Hong et al., 2015). Alternatively, focal adhesions, which are complex multimolecular protein assemblies, are needed for VSMC contractility and provide mechanical stability to the blood vessel (Min et al., 2012; Ribeiro-Silva et al., 2021). The stability of such focal adhesion complexes is affected by external force and reorganized in response to stretch (Kong et al., 2008; De, 2018). Therefore, it can be speculated that acutely altered pulsatile stretching conditions modulate aortic focal adhesion stability, which would lead to changes in mechanical properties of the aorta. Indeed, our previous data indicated that an acute increase in pulsatile load decreased arterial rigidity and VSMC contractility (Neutel et al., 2021). However, how such acute changes in cyclic stretch affect focal adhesion function and thereby modulate aortic biomechanical properties (i.e., viscoelasticity) remains unclear.

In the present study, we used the classic Kelvin-Voigt model, supplemented with an additional inertial component, to study viscoelasticity of murine aortic tissue *ex vivo*. First, we determined regional differences in viscoelastic properties between thoracic and abdominal aortic tissues. Secondly, we investigated how viscoelasticity can be modulated by both cyclic stretch and VSMC contraction along with the interaction between both. Special attention was given to VSMC focal adhesion, since it acts as a bi-directional bridge between cell behavior and tissue viscoelasticity (Hui et al., 2021).

## 2 Methods

### 2.1 Animals

Male C57BL/6J mice (6 months old; Charles River Laboratories, France) were housed in the animal facility of the University of Antwerp in standard cages with 12 h–12 h light-dark cycles and had free access to regular chow and tap water. The animals were euthanized by perforating the diaphragm while under anesthesia [sodium pentobarbital (Sanofi, Belgium), 75 mg/kg i.p.]. The ascending, descending and infrarenal aorta were carefully removed and stripped of adherent tissue. The tissue was cut into segments of 2 mm. The segments were immersed in Krebs Ringer (KR) solution (37°C, 95% O2/5% CO2, pH 7.4) containing (in mM): NaCl 118, KCl 4.7, CaCl2 2.5, KH2PO4 1.2, MgSO4 1.2, NaHCO3 25, CaEDTA 0.025 and glucose 11.1. The study was waived by the Ethics Committee for Animal Research at the University of Antwerp according to article 3 of the EU legislation (L 276/38, 2010).

### 2.2 Measuring ex vivo arterial biomechanics: Rodent oscillatory tension setup to measure arterial compliance (ROTSAC)

To determine stiffness of aortic vessel segments and their viscoelastic properties (*ex vivo*), an in-house developed setup termed ROTSAC was used (Leloup et al., 2016). In brief, aortic segments of 2 mm were mounted between two parallel wire hooks in organ baths of 10 mL. Force and displacement of the upper hook were controlled and measured with a force-length transducer. The segments were continuously oscillating between alternating preloads at a frequency of 10 Hz. Calibration of displacement of the upper hook allowed the calculation of the vessel diameter. The Laplace relationship was used to calculate the transmural pressure from the distension force and vessel dimensions:

$$P=\frac{F}{l.D}$$

With *F* the force, *l* the length and *D* the diameter of the vessel segment. While pressure is not generated in the ROTSAC setup, the calculated pressure has been shown to be a relevant parameter and has been successfully used in previous research, with good translation to *in vivo* PWV measurements in mice (De Moudt et al., 2022). The stiffness of the aortic segments were quantified through the Peterson’s pressure-strain modulus of elasticity (Ep). The Ep was calculated as follows:

$$E_{p}=D_{0}.\frac{∆P}{∆D}$$

With D_0_ as the diastolic diameter, ∆D the distension and ∆P the pulse pressure. Additionally, strain rate, which is the change in strain with respect to time, was determined in some set of experiments and was calculated as:

$$Strain\;rate=\frac{\left(\frac{∆D}{D_{0}}\right)}{∆t}$$

With ∆D the difference between the maximum systolic diameter and the minimum diastolic diameter, D_0_ as the diastolic diameter and ∆t the time between max systolic diameter and minimum diastolic diameter.

### 2.3 *Ex vivo* analysis of viscoelastic properties of aortic segments

The viscoelastic behavior of aortic segments was studied under different conditions. An adaptation to the Kelvin-Voigt model as described by Bia et al. (Bia et al., 2005a) was applied to analyze the viscoelastic properties of the aortic segments. This model separates the elastic and viscous component so that the total pressure-diameter relationship (P_total_) can be described through the following formula:

$$P_{Total}=P_{Elastic}+P_{Viscous}$$ (1)

With P_elastic_ and P_viscous_ being the elastic and viscous pressure-diameter relationships respectively. An inertial component was added to further optimize the model so that Eq. 1 can be written as:

$$P_{Total}=P_{Elastic}+P_{Viscous}+P_{Inertial}$$ (2)

The first derivative of the diameter in function of time represents the P_viscous_, whereas the second derivative of the diameter in function of time represents the P_inertial_. Therefore, Eq. 2 can be re-written as:

$$P_{Elastic}=P_{Total}-\left(E_{n}.\;\frac{dD}{dt}+E_{M}.\;\frac{d^{2}D}{dt^{2}}\right)$$ (3)

**FIGURE 1:**
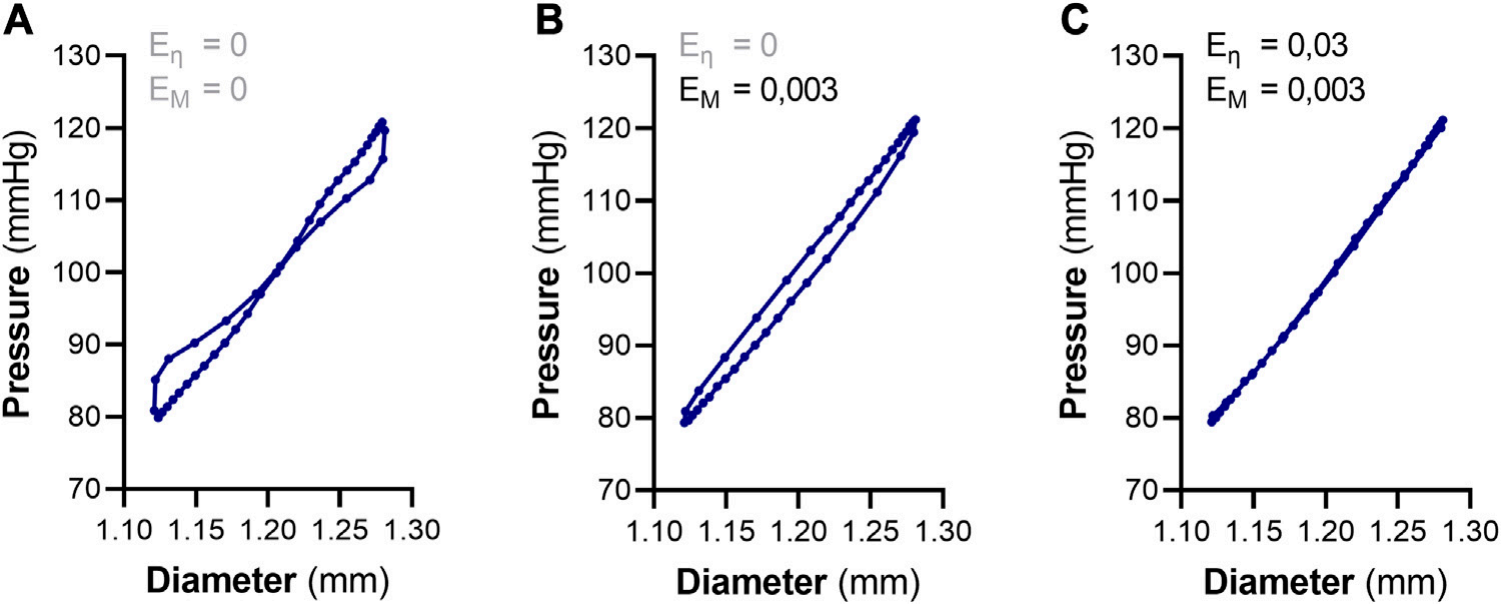
Analyzing viscoelastic properties of aortic segments using pressure-diameter tracings, generated from the ROTSAC setup. **(A)** A raw “pressure-diameter” tracing of a single distension cycle of an aortic segment between 80 and 120 mmHg. The tracing depicts a viscous component (i.e., hysteresis) as well as an inertial component. **(B)** Removal of the inertial component of the raw tracing by manual iteration of the inertial modulus (E_M_). This results in a pressure-diameter curve with clear hysteresis. **(C)** Removal of the viscous component (/hysteresis) by manual iteration of the viscous modulus (E_η_) after having removed the inertial component. When the minimum area of hysteresis is reached, a pure elastic pressure-diameter is obtained. The slope at 100 mmHg of the resulting curve is considered as the elastic modulus (E_E_). The values of E_M_ and E_η_ at the minimum area of hysteresis are measures of the total amount of inertia and wall viscosity, respectively.

Were E_η_ and E_M_ are the viscous and inertial modulus, respectively (Eq. 3). For each “raw” pressure-diameter tracing obtained from the ROTSAC, the viscous and inertial modulus were manually iterated to obtain the minimum area of hysteresis (Figure 1). The corresponding E_η_ value was considered to be a measure of the amount of hysteresis in the sample. Afterwards, the slope of the resulting, elastic, pressure-diameter relationship was considered as the elastic modulus (E_E_). Of note, the elastic modulus should not be confused with the term “elasticity”, as the latter refers to the flexibility/distensibility of tissue, whereas an increase in the elastic modulus denotes a higher rigidity.

### 2.4 Histological evaluation of elastin tortuosity and nucleus circularity

Aortic segments were fixated with 4% formaldehyde (PBS buffered) under cyclic stretch in a ROTSAC setup at 80 and 120 mmHg diastolic and systolic pressure, respectively. After 30 min, the aortic segments were transferred from the organ baths to well plates with 4% formaldehyde (PBS buffered). After 24 h fixation, segments were transferred to well plates with 60% isopropanol followed by paraffin-embedding. Transversal sections (5 µm) were stained with hematoxylin-eosin (H&E). Eosin amplifies the green fluorescence emitted by elastin fibers (autofluorescence). Hence, elastin fibers could be identified using a CELENA S fluorescence microscope (×20 objective). Of note, one transversal section per aortic segment was subjected to immunohistochemical staining and consequent analysis. Tortuosity of elastin fibers was quantified in ImageJ as the L/L0 ratio, with L the actual, “wavy” length of the fiber from point A to B and L0 the, straight, distance between point A and B. Nucleus circularity was measured on the same histological samples as used for analyzing elastin fiber tortuosity. The analysis was conducted in Fiji as well; the DAPI signal (i.e., the nucleus) was extracted and its circularity was measured using the “Analyze particles” function.

### 2.5 Experimental protocol(s)

To study the effect of cyclic stretch on aortic viscoelasticity, two experiments were conducted. In a first experiment, both the mean and pulse pressure were kept constant (i.e. 100 mmHg mean pressure and 40 mmHg pulse pressure) while the pulse frequency was altered (ranging from 1 Hz to 25 Hz). Of note, supra-physiological frequencies (i.e. 25 Hz) were used to gain a better understanding of the *ex vivo* biomechanical properties of the aorta. However, it needs to be kept in mind that such frequencies are not relevant *in vivo*. The viscoelastic parameters were quantified at each frequency. In a second experiment, both the mean pressure and the pulse frequency were kept constant (i.e. 100 mmHg mean pressure at a pulse frequency of 10 Hz), while the pulse pressure was altered (ranging from 10 mmHg to 100 mmHg). Thoracic ascending, thoracic descending and abdominal infrarenal aortic tissues were tested in parallel. These experiments were repeated in the presence of PP2 (Merck Life Sciences, P0042) and Cytochalasin D (Merck Life Sciences, C8273) to study the role of VSMC adhesion in pulsatility-modulated viscoelasticity.

Further, the effect of cyclic stretch on VSMC contraction-mediated viscoelasticity was investigated. Aortic segments were pre-contracted with 2 µM phenylephrine to induce VSMC contraction under cyclic stretch in a ROTSAC (80–120 mmHg, diastolic and systolic pressures respectively, 10 Hz). Thirty minutes after phenylephrine administration, systolic pressure was increased from 120 to 170 mmHg. After 4 minutes, the systolic pressure was brought back to 120 mmHg. Diastolic pressure was kept constant at 80 mmHg during the high systolic pressure bout. Viscoelastic parameters were calculated 1 minute after returning to normal systolic pressure. Additionally, elastin fiber tortuosity was measured on aortic samples, fixed under cyclic stretch, 1 minute after the high pulsatile bout.

### 2.6 Western blotting

To study the effect of pulsatility and VSMC contraction on focal adhesion kinase (FAK), aortic tissues were collected and mounted in a ROTSAC as described above. Phenylephrine (2 µM) was administered to elicit VSMC contraction under cyclic stretch. Fifteen minutes after inducing VSMC contraction, aortic segments were removed from the ROTSAC and collected in Laemmli sample buffer (Bio-Rad), supplemented with β-mercaptoethanol (5%), to facilitate cell lysis. Samples were heat-denaturized for 5 min at 100°C. Alternatively, to study the different focal adhesion protein composition in the abdominal infrarenal aorta compared to the thoracic descending aorta, the full thoracic descending aorta and the full abdominal infrarenal aortic tissue were collected from individual mice. Samples were homogenized in RIPA buffer containing protease and phosphatase inhibitors. Protein concentrations were determined using the BCA method. Samples were then diluted in Laemmli sample buffer (Bio-Rad) containing 5% β-mercaptoethanol (Sigma-Aldrich) and heat-denaturized for 5 min at 100°C.

Samples were loaded on Bolt 4%–12% bis-tris gels (Invitrogen) for gel electrophoresis, followed by wet transfer on polyvinylidene fluoride membranes. Membranes were blocked in Odyssey^®^ Blocking Buffer (Li-Cor Bioscience) and probed with primary antibody (overnight, 4°C). The following primary antibodies and dilutions were used: 1:1000 rabbit anti-FAK (phosphorylated at Tyr397) (Abcam, ab81298), 1:5000 mouse anti-β-actin (Abcam, ab8226), 1:1000 rabbit anti-FAK (Abcam, ab40794), 1:1000 rabbit anti-vinculin (Abcam, ab155120), 1:1000 rabbit anti-Paxillin (Abcam, ab32084), 1:2000 mouse anti-Talin1 (Abcam, ab108480), 1:5000 mouse anti-b-actin (Abcam, ab8226), 1:5000 rabbit anti-b-actin (Abcam, ab115777). Subsequently, (IR)-conjugated secondary antibodies (anti-rabbit: IgG926-32211 and anti-mouse: IgG926-68070; Li-Cor Biosciences) were used for IR fluorescence detection using an Odyssey SA infrared imaging system (Li-Cor Biosciences). Western blot signal was analyzed using Image Studio Lite (LI-COR). The western blot panels pictured in this study are derived from one single blot and represent the mean values of conditions used.

### 2.7 Proximity ligation assay

To visualize the binding interaction of talin and vinculin, a Duolink^®^ Proximity Ligation Assay (PLA, Merck Life Sciences, DUO92105) was performed. In brief, aortic segments were fixated under cyclic stretching conditions with 4% formaldehyde (PBS buffered) and transferred to isopropanol for paraffin-embedding. Afterwards, an immunohistochemical staining was performed on transversal sections with anti-Talin1 (Merck Life Sciences, SAB2501307) and anti-vinculin (Abcam, ab155120) primary antibodies. Next, secondary antibodies, conjugated with oligonucleotides, targeted against the used primary antibodies were administered. Duolink^®^ ligation and amplification solutions were added according to the manufacturer’s protocol. When two proteins are within 40 nm of each other, the PLA produces a fluorescent spot. After the PLA staining, the samples were visualized under a CELENA S fluorescence microscope (×40 magnification) to visualize these fluorescent spots. The Duolink^®^ PLA fluorescent spots in the medial layer of aortic tissue were counted using ImageJ software and normalized to the number of cells in the tunica media.

### 2.8 Statistics

**FIGURE 2:**
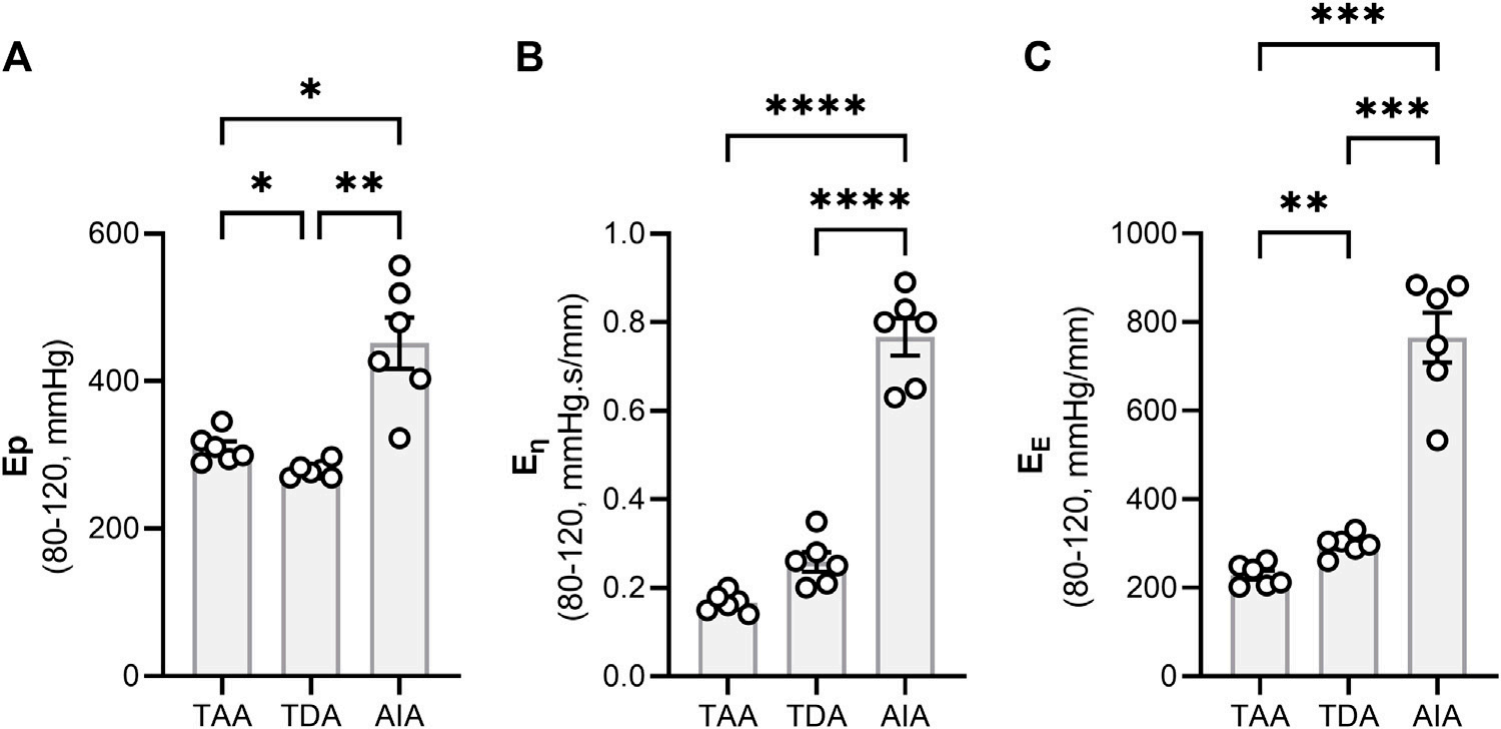
The viscoelastic properties of central aortic tissue. The viscoelastic properties of central aortic tissue were determined via an adapted Kelvin-Voigt model using pressure-diameter tracings as described in the method section. There were no significant differences in **(A)** overall stiffness (quantified using the Peterson’s elastic modulus) and **(B, C)** viscoelastic properties between the thoracic ascending and thoracic descending aorta. However, the abdominal infrarenal aorta had a significantly higher Ep as compared to both thoracic tissues and had also a significantly higher viscous and elastic modulus. Statistical analysis was performed by using a Repeated Measures One-way ANOVA with a Tukey *post hoc* test for multiple comparisons (n = 6). *p < 0.05, **p < 0.01, ***p < 0.001, ****p < 0.0001. Ep = Peterson’s Modulus of Elasticity; TAA = Thoracic Ascending Aorta; TDA = Thoracic Descending Aorta; AIA = Abdominal Infrarenal Aorta.

**FIGURE 3:**
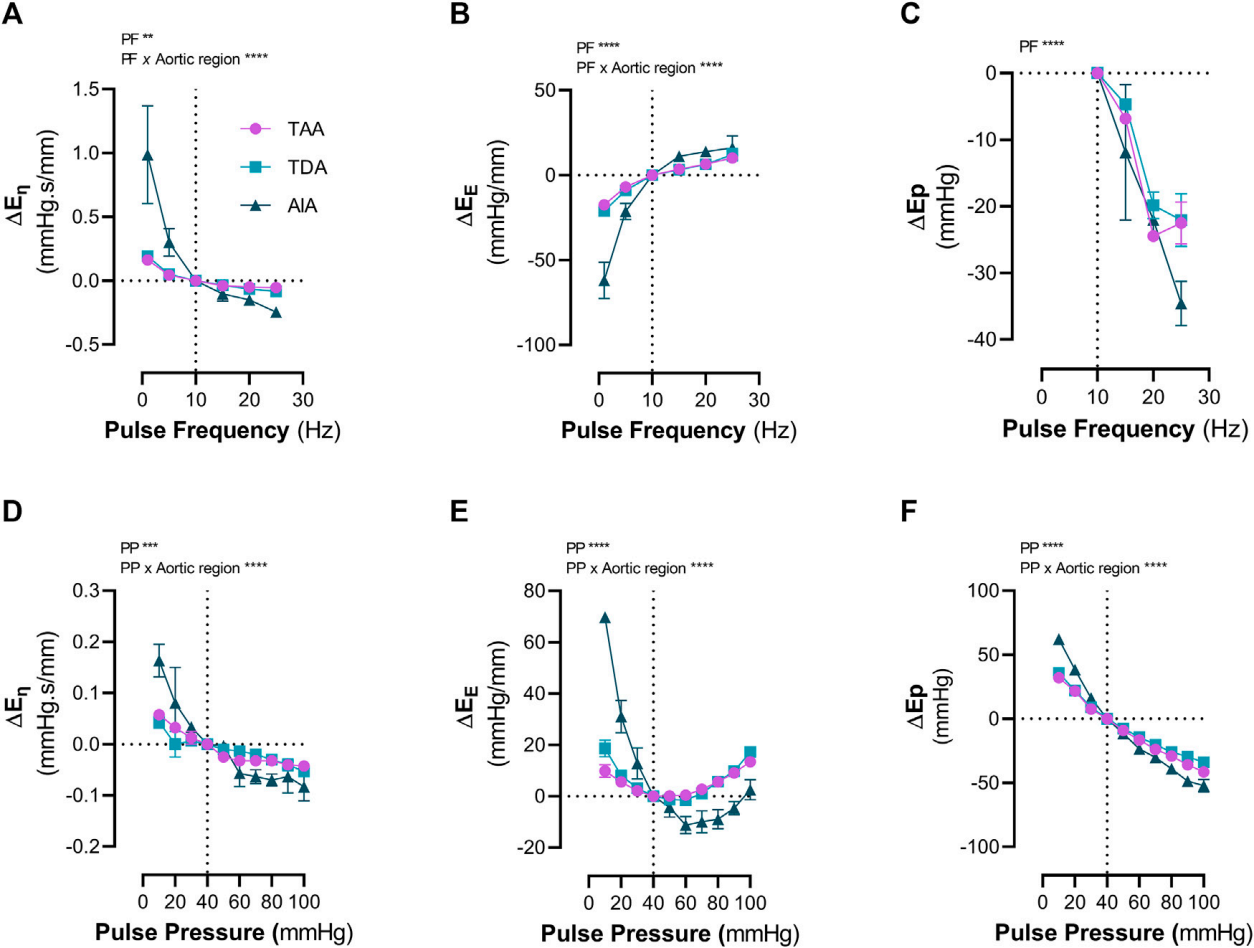
Increased cyclic stretch, either through pulse pressure or pulse frequency, modulates aortic viscoelasticity. The changes in viscoelastic properties due to altered cyclic stretch were measured in aortic segments from the thoracic ascending aorta (TAA), thoracic descending aorta (TDA) and the abdominal infrarenal aorta (AIA). Increasing the pulse frequency from 1 Hz to 25 Hz (100 mmHg mean pressure, 40 mmHg pulse pressure) decreased **(A)** the viscous modulus, increased **(B)** the elastic modulus and decreased **(C)** the Ep in all types of aortic segments. Increasing the pulse pressure from 10 mmHg to 100 mmHg (100 mmHg mean pressure, 10 Hz) decreased **(D)** the viscous modulus. **(E)** A biphasic response of the elastic modulus was observed when increasing pulse pressure from 10 mmHg to 100 mmHg. **(F)** Ep decreased with increasing pulse pressure. Statistical analysis was performed using a Two-Way ANOVA with a Tukey *post hoc* test for multiple comparisons. n = 4 per group. **p < 0.01, ***p < 0.001, ****p < 0.0001. Ep = Peterson’s Modulus of Elasticity; Eη = Viscous Modulus; EE = Elastic Modulus; TAA = Thoracic Ascending Aorta; TDA = Thoracic Descending Aorta; AIA = Abdominal Infrarenal Aorta.

**FIGURE 4:**
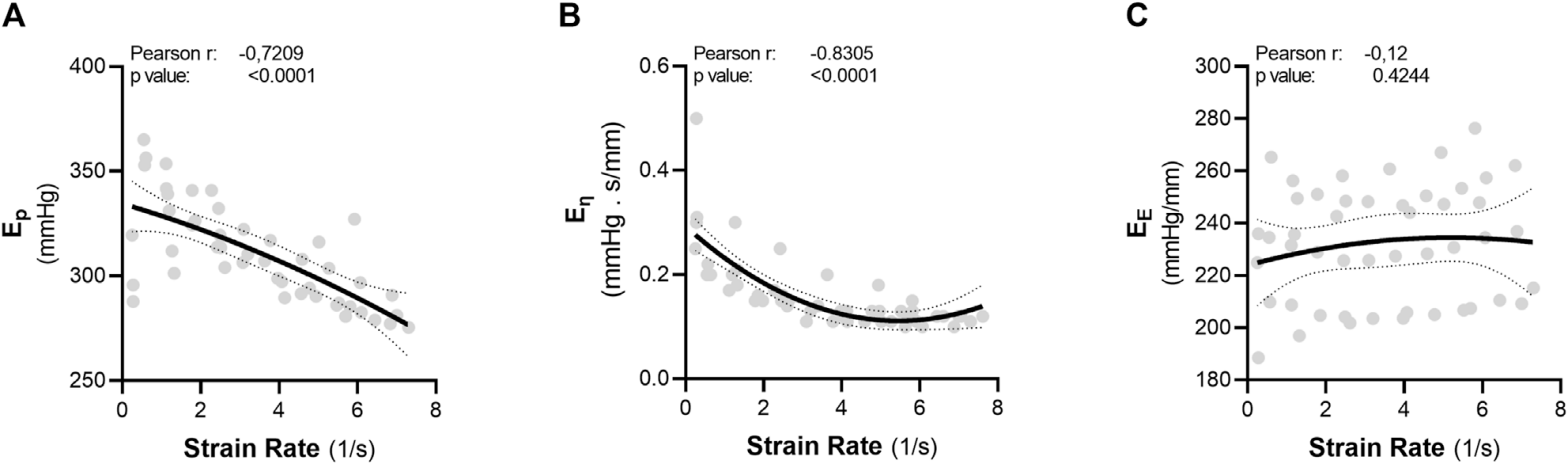
Strain rate is inversely correlated with aortic viscosity in the thoracic ascending aorta. The parameters “pulse frequency” and “pulse pressure” were each transformed to another parameter: strain rate. Strain rate was calculated as the change in diameter from diastolic pressure to systolic pressure, divided by Dt. There was an inverse relationship between strain rate and both **(A)** Ep and **(B)** the viscous modulus. There was no correlation between strain rate and **(C)** the elastic modulus. Statistical analysis: Pearson correlation. Ep = Peterson’s Modulus of Elasticity; E_η_ = Viscous Modulus; E_E_ = Elastic Modulus.

**FIGURE 5:**
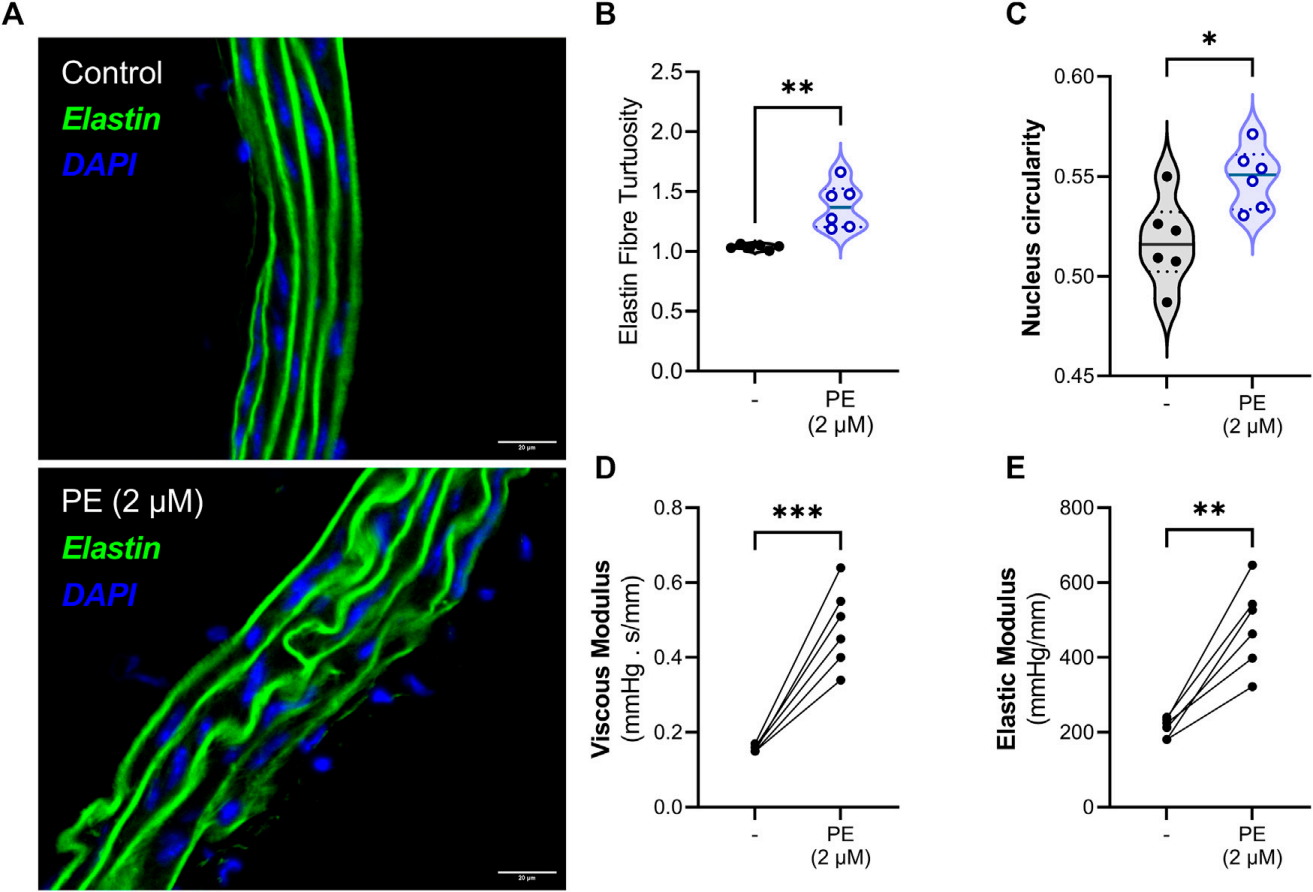
Vascular smooth muscle cell contraction induces elastin fibre tortuosity and increases aortic viscoelasticity. **(A, B)** Indeed, VSMC contraction increases the tortuosity of elastin fibers in the medial layer of the aorta. **(C)** Additionally, the circularity of the nuclei is increased after VSMC contraction. Finally, acute contraction with phenylephrine increases both **(D)** the viscous and the **(E)** elastic properties of the aortic segment. Statistical analyses: **(B–C)** Unpaired T-test, **(D–E)** paired T-test. **(B–E)** n = 6. *p < 0.05, **p < 0.01, ***p < 0.001. **PE** = phenylephrine.

**FIGURE 6:**
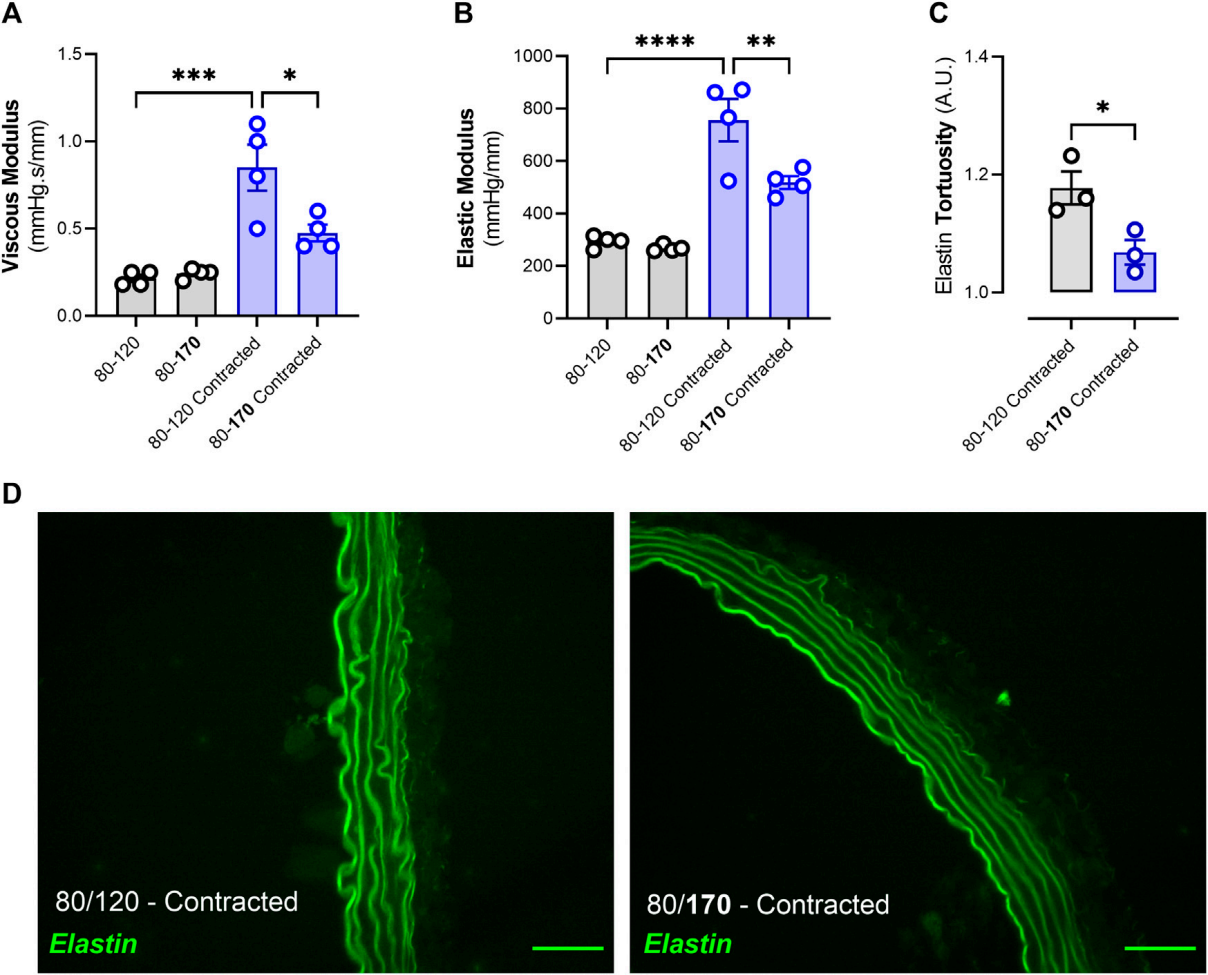
Acute high pulsatile bout decreases viscoelastic properties and decreases elastin tortuosity in pre-contracted aortic segments. Two out of four aortic segments (originating from the same mouse) were pre-contracted with 2 µM phenylephrine while subjected to high frequency cyclic stretch (80–120 mmHg). After 30 min, one contracted and one uncontracted segment were subjected to a high pulsatile bout for 4 minutes (increase in systolic pressure to 170 mmHg). VSMC contraction-increased viscous and elastic moduli were attenuated after an acute high pulsatile bout **(A, B)**. Furthermore, VSMC-contraction induced elastin fiber tortuosity was also decreased after an acute high pulsatile bout **(C, D).** Statistical analyses; **(A–B)** One-way ANOVA with a Tukey *post hoc* test for multiple comparisons, n = 4; **(C)** Unpaired T-test, n = 3. *p < 0.05, ***p < 0.001, ****p < 0.0001.

**FIGURE 7:**
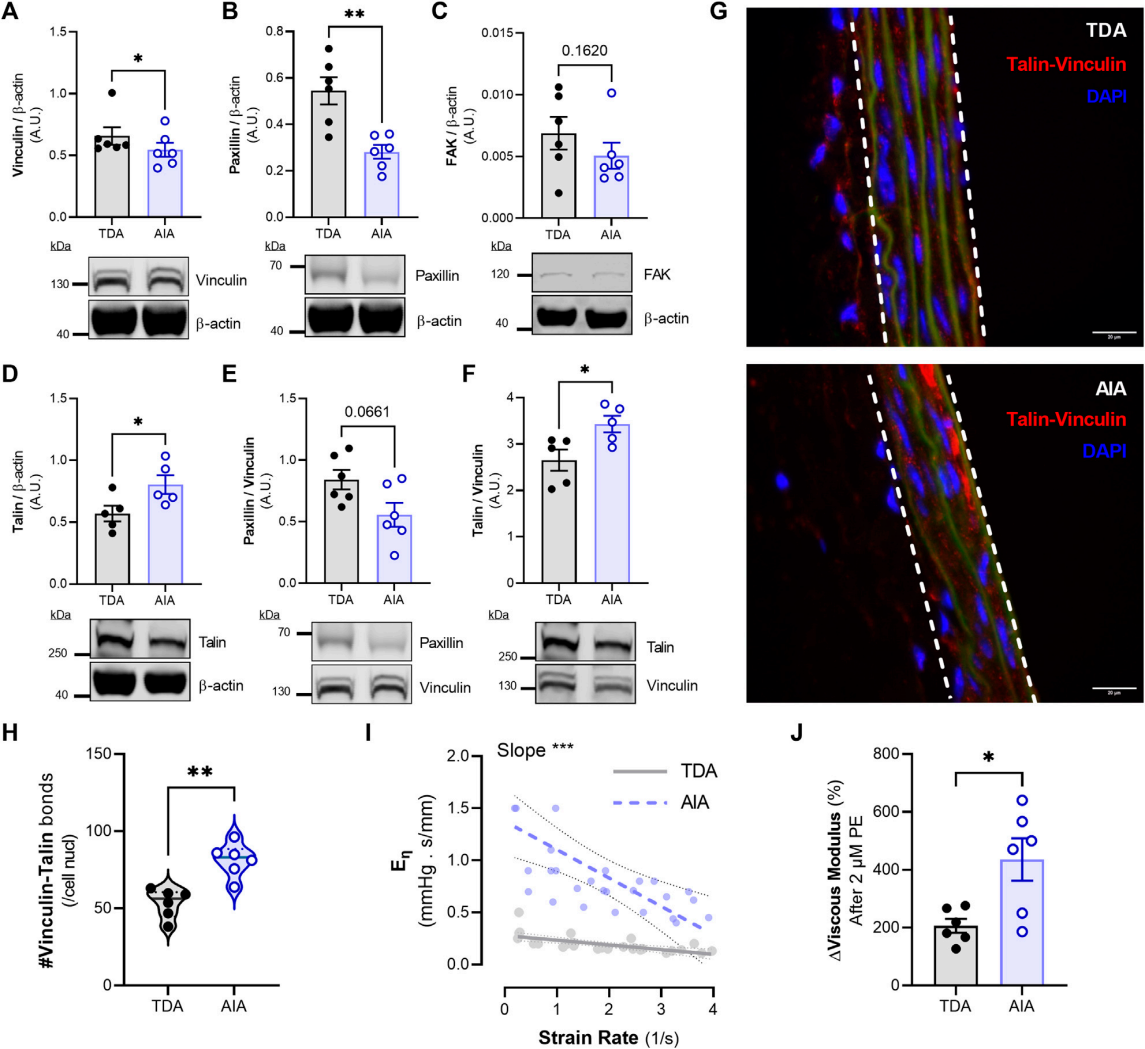
Composition of focal adhesion proteins along the thoracic and abdominal aorta and the differential cyclic stretch- and contraction-mediated viscoelastic responses. The thoracic descending and the abdominal infrarenal aorta were excised from mice and cleaned from adipose tissue. Afterwards, a series of immunoblots were performed for different focal adhesion proteins **(A–F)**. Furthermore, a proximity ligation assay was performed to analyze the amount of medial talin-vinculin bonds **(G, H).** The white lines denote the medial layer of the aortic sections. Scale bar = 20 µm. Lastly, viscoelastic responses to strain rate **(I)** and smooth muscle cell contraction **(J)** were compared between the TDA and the AIA. Statistical analyses: **(A–H, J)** paired Student’s t-test, n = 5-6 per group; **(I)** Least squares regression plus extra sum-of-squares F test to compare best fit values (i.e., the slope). *p < 0.05, **p < 0.01, ***p < 0.001. TDA = Thoracic Descending Aorta; AIA = Abdominal Infrarenal Aorta; E_η_ = Viscous Modulus.

**FIGURE 8:**
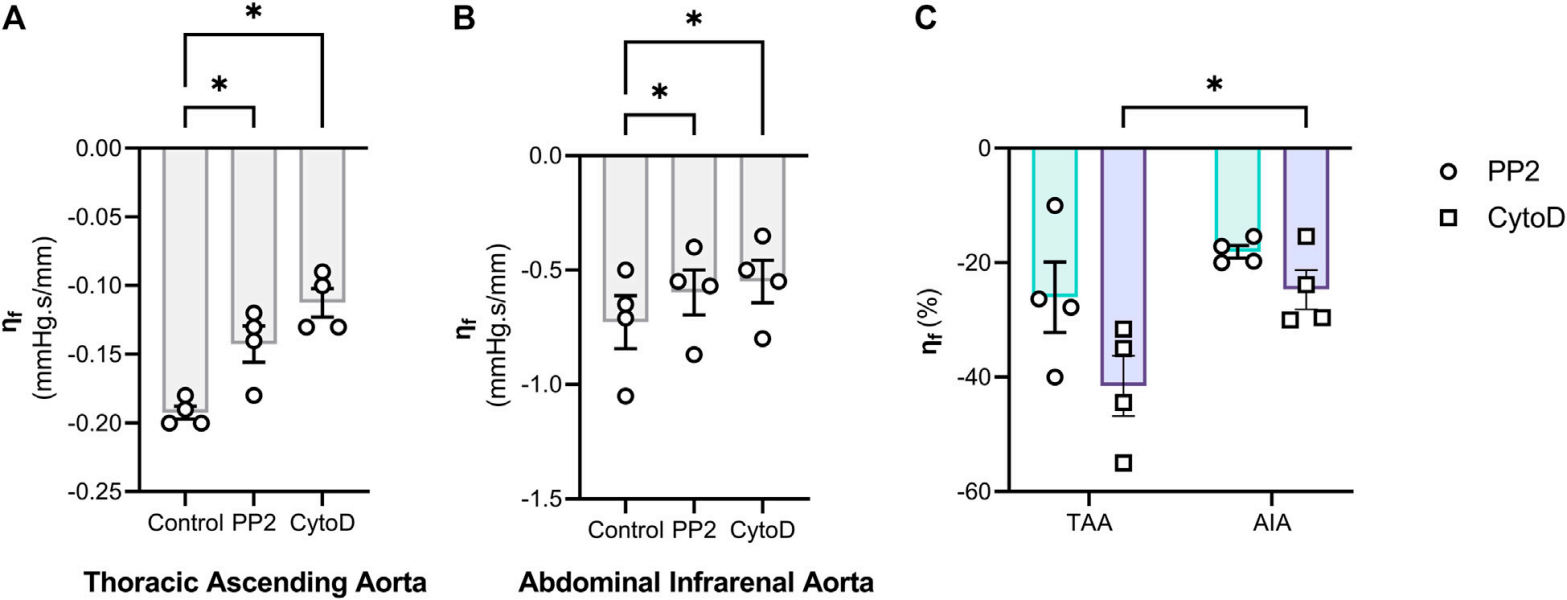
Disruption of either Focal adhesion activity or the F-actin cytoskeleton integrity attenuates the frequency-mediated changes in viscous properties. The role of focal adhesion and F-actin was further investigated by treating thoracic ascending aorta (TAA) **(A)** and abdominal intrarenal aorta **(B)** segments with 10 µM PP2 or 10 µM Cytochalasin D. Afterwards, pulse frequency was increased from 1 Hz to 25 Hz at 100 mmHg mean pressure (80/120 mmHg, diastolic and systolic pressure). The difference in viscous modulus between 25 Hz and 1 Hz (denoted as “η_f_”) was smaller after either treatment, indicating that focal adhesion and F-actin are mechanosensors which are sensitive to pulse frequency. The relative effect of PP2 and Cytochalasin D on η_f_ are shown in **(C)**. Statistical analysis: **(A, B)** repeated measures One-Way ANOVA with a Holm-Sidak *post hoc* test for multiple comparisons; **(C)** Two-way ANOVA with a Sidak *post hoc* test for multiple comparisons. n = 4. *p < 0.05. **η**_**f**_ = Frequency-mediated reduction in the viscous modulus (=E_η__25 Hz_–E_η__1Hz_); E_η_ = Viscous Modulus; TAA = Thoracic ascending aorta; AIA = Abdominal infrarenal aorta.

Statistical analyses were performed in GraphPad Prism 9.5.0. All data are presented as mean ± SEM. Dots represent n samples from independent experiments. To compare viscoelasticity along the aortic tree, a repeated measures one-way ANOVA (with a Tukey *post hoc* test for multiple comparisons) was performed to compare the thoracic ascending, thoracic descending and abdominal infrarenal aorta (Figure 2). Furthermore, a one-way ANOVA test (with a Tukey *post hoc* test for multiple comparisons) has also been used to compare the viscous/elastic modulus before and after a high pulsatile bout in the presence or absence of a phenylephrine (Figures 6A, B). Lastly, to compare the effect of PP2 and cytochalasin D on aortic viscoelasticity, another one-way ANOVA with a Sidak *post hoc* test for multiple comparisons was used (Figure 8). Further, to assess the effect of either pulse pressure or pulse frequency on aortic tissue viscoelasticity and the comparison between different aortic regions, a two-way ANOVA was performed (Figure 3). A Pearson correlation test was used to analyse the relationship between strain rate and aortic viscoelasticity (Figure 4). (Un)paired T-test(s) have been used for: comparing elastin fibre tortuosity before and after VSMC contraction (Figure 5), the viscous/elastic modulus before and after VSMC contraction (Figure 5), comparing elastin fibre tortuosity before and after an acute high pulsatile bout (Figure 6C), analysing differences in focal adhesion proteins between tissue lysates from the thoracic descending aorta and the abdominal infrarenal aorta (Figures 7A–G), comparing the amount of talin-vinculin bounds between the thoracic descending and abdominal infrarenal aorta (Figure 7H), comparing *ex vivo* elastin fiber tortuosity between the thoracic descending aorta and the abdominal infrarenal aorta (Supplementary Figure S3). A Least squares regression plus extra sum-of-squares F test to compare best fit values has been used to compare the relationship between strain rate and the viscous modulus between the thoracic descending aorta and the abdominal infrarenal aorta (Figure 7I). To compare the effect of VSMC contraction, pulse pressure and/or pulse frequency on FAK phosphorylation, one-sample T-tests (hypothetical value = 0) were performed on the Log_2_ transformed western blot data (Supplementary Figure S2). Statistical tests are specified in the figure legends. Differences were considered significant when *p* < 0.05.

## 3 Results

### 3.1 Viscoelasticity in the central aortic tree increases along the distal aorta

Aortic segments from the central aorta were subjected to high frequency cyclic stretch in a ROTSAC at a diastolic and systolic pressure of 80 and 120 mmHg, respectively. Separating the viscous and elastic properties of aortic segments of distinct anatomical regions across the central aorta revealed both an increased stiffness as well as increased viscoelasticity in the distal aorta (Figure 2). The abdominal infrarenal aorta (AIA) had a significantly higher isobaric stiffness as compared to the thoracic ascending aorta (TAA) (*p* < 0.05) and thoracic descending aorta (TDA) (*p* < 0.01), whereas the TAA had a significantly higher Ep than the TDA (*p* < 0.05) (Figure 2A). Additionally, the AIA had a significantly higher viscous modulus than the TAA (*p* < 0.0001) and TDA (*p* < 0.0001) (Figure 2B). No differences were observed in the viscous modulus between the TAA and the TDA. Finally, the elastic modulus was significantly higher in the AIA than in the TAA (*p* < 0.001) and TDA (*p* < 0.001), whereas the TDA had a significantly (*p* < 0.01) higher elastic modulus than the TAA (Figure 2C).

### 3.2 Increased cyclic stretch, either through increased pulse pressure or pulse frequency, decreases aortic viscoelasticity

We further investigated how pulse pressure and pulse frequency affect aortic viscous and elastic properties in different aortic regions under basal, uncontracted conditions. Increasing pulse frequency significantly decreased both the viscous modulus (*p* < 0.01) and the Peterson’s modulus (*p* < 0.0001), whereas it significantly increased the elastic modulus (*p* < 0.0001) (Figures 3A–C). Moreover, there was a significant interaction between the pulse frequency and aortic tissue type for the viscous and elastic modulus (*p* < 0.0001 and *p* < 0.0001, respectively). Increasing the pulse pressure significantly decreased both the viscous modulus (*p* < 0.0001) and the Peterson’s modulus (*p* < 0.0001), whereas a significant (*p* < 0.0001) biphasic response was observed for the elastic modulus (Figures 3D–F). A significant interaction was also observed between the pulse pressure and tissue type for the viscous modulus (*p* < 0.0001), elastic modulus (*p* < 0.0001) and the Peterson’s modulus (*p* < 0.0001).

### 3.3 Strain rate is inversely correlated with the viscous properties of aortic tissue

A common factor that is shared by increasing either pulse pressure or pulse frequency *ex vivo* is an increase in strain rate (Supplementary Figure S1). Therefore, the pulse pressure and pulse frequency parameters from the abovementioned dataset (Figure 3) were transformed to strain rate. Afterwards, the data was merged to identify whether strain rate is a common factor in regulating viscoelasticity. In the ascending aorta, there is a significant, inverse, correlation between strain rate and both the Peterson’s modulus (r = −0.7209, *p* < 0,0001) and the viscous modulus (r = −0.8305, *p* < 0.0001) (Figures 4A, B). However, there was no correlation between strain rate and the elastic modulus (Figure 4C).

### 3.4 Vascular smooth muscle cell contraction increases both elastin tortuosity and viscoelastic properties

The role of VSMC contraction in modulating aortic viscoelasticity was investigated by eliciting maximum α1-adrenoreceptor-mediated stimulation with phenylephrine (PE) in thoracic descending aortic tissue. Histological evaluation of aortic segments, incubated for 30 min with PE in a ROTSAC, revealed a significantly (*p* < 0.01) increased tortuosity of the medial elastin fibers after VSMC contraction (Figures 5A, B). Moreover, VSMC contraction significantly (*p* < 0.05) increased (medial cell) nucleus circularity (Figures 5A, C). Measuring viscoelastic parameters before and after 2 μM PE administration revealed a significant increase in both the viscous (*p* < 0.001) and elastic moduli (*p* < 0.01) due to VSMC contraction (Figures 5D, E).

### 3.5 An acute high pulsatile bout decreases contraction-enhanced viscoelasticity and reverses elastin tortuosity

Thoracic descending aortic segments were pre-contracted with 2 µM phenylephrine combined with 300 μM N-Ω-Nitro-L-arginine methyl ester hydrochloride (L-NAME). L-NAME was added to suppress nitric oxide release and to facilitate maximum VSMC contraction. After 30 min, systolic pressure was increased from 120 mmHg to 170 mmHg for 4 min, after which the systolic pressure was re-adjusted to 120 mmHg. Viscoelasticity was measured 1 minute after returning to baseline diastolic and systolic pressures. Whereas the acute pulsatile bout did not affect uncontracted aortic segments, 4 min of high systolic pressure significantly (*p* < 0.05) decreased both the viscous and elastic modulus of pre-contracted segments (Figures 6A, B). Furthermore, elastin fiber tortuosity was measured on aortic segments (formaldehyde-fixed under cyclic stretch) 1 minute after the acute pulsatile bout. High systolic pressure significantly (*p* < 0.05) decreased elastin fiber tortuosity when returning to 80 and 120 mmHg diastolic and systolic pressure, respectively (Figures 6C, D).

### 3.6 Cyclic stretch frequency inhibits phenylephrine-induced FAK phosphorylation (Tyr397)

To further determine the effect of cyclic stretch on de-stiffening of aortic segments, a series of immunoblots were performed for focal adhesion kinase (FAK). First, the effect of acute changes in pulse pressure on FAK were investigated by increasing the systolic pressure from 120 mmHg to 170 mmHg for 4 min on pre-contracted segments. Phenylephrine (2 μM, combined with 300 μM L-NAME) significantly increased (*p* < 0.05) FAK phosphorylation at Tyr397 at normal, physiological pressures (80–120 mmHg) (Supplementary Figure S2). One minute after an acute high pulsatile bout (a systolic pressure of 170 mmHg for 4 min), no change in FAK phosphorylation was observed (*p* = 0.07) in contracted segments. Additionally, the pulsatile bout did not alter FAK phosphorylation in uncontracted segments. Secondly, the effect of pulse frequency on FAK phosphorylation was investigated. Instead of subjecting aortic segments to a “pulsatile” bout (i.e., an increase in systolic pressure), (un)contracted aortic segments were subjected to a “frequency bout” (i.e., pulse frequency was changed from 10 Hz to either 1 Hz or 25 Hz for 4 minutes). Western blots revealed that VSMC contraction-induced FAK phosphorylation (Tyr 397) was absent after changing the pulse frequency to either 1 Hz or 25 Hz (Supplementary Figure S2).

### 3.7 Elastin fibers in infrarenal aortic tissue are tortuous in basal conditions

Aortic segments from the thoracic descending aorta (TDA) and the abdominal infrarenal aorta (AIA) were fixed in 4% formaldehyde while oscillating between 80 and 120 mmHg diastolic and systolic pressure, respectively (with a pulse frequency of 10 Hz). The autofluorescence of elastin fibers was visualized on cross-sections using a fluorescence microscope (Supplementary Figure S3A). The elastin fibers in the infrarenal aorta were significantly (*p* < 0.05) more tortuous than the elastin fibers in the thoracic descending aorta in uncontracted, isobaric, stretched, conditions (Supplementary Figure S3B). No differences were observed in nucleus circularity between the medial cells from the thoracic descending aorta and the abdominal infrarenal aorta (Supplementary Figure S3C).

### 3.8 Differential focal adhesion composition in the infrarenal aorta is associated with altered VSMC contraction and cyclic stretch-modulated viscoelastic properties

Because we have associated elastin fiber tortuosity with activity from the focal adhesion complex, we evaluated focal adhesion activity in the AIA in basal uncontracted conditions. We performed a series of immunoblots for focal adhesion proteins in aortic tissue from the AIA and the TDA. There was a significantly (*p* < 0.05) increased amount of vinculin in the TDA compared to the AIA (Figure 7A). Furthermore, there was significantly (*p* < 0.01) more paxillin in the TDA than in the AIA (Figure 7B). No differences in FAK were observed between the TDA and the AIA (Figure 7C). Talin levels were significantly (*p* < 0.05) increased in the AIA compared to the TDA (Figure 7D). Since vinculin binds to both paxillin and talin, enabling mechanical coupling between the ECM and the cytoskeleton, we evaluated the paxillin/vinculin and talin/vinculin ratios. Interestingly, whereas the paxillin/vinculin ratio was not significantly different between the TDA and the AIA. The talin/vinculin ratio was significantly (*p* < 0.05) higher in the AIA (Figures 7E, F). We next determined if there was a higher level of focal adhesion maturation present in the AIA compared to the TDA (determined as the active amount of talin-vinculin bonds in aortic tissue). In order to measure this, we performed a “Proximity Ligation Assay”, for talin and vinculin, on aortic tissue that was formaldehyde-fixed under stretch in a ROTSAC (Figure 7G). Interestingly, there was a significantly (*p* < 0.01) higher amount of talin-vinculin bonds in the medial layer of the AIA compared to the TDA (Figure 7H). Furthermore, we explored if the viscoelastic response to VSMC contraction and cyclic stretch were more pronounced in the AIA, compared to the TDA. Indeed, the (inverse) relationship between strain rate and the viscous modulus was significantly (*p* < 0.001) more pronounced in the AIA, as indicated by an increased slope after non-linear regression analysis (Figure 7I). Finally, eliciting VSMC contraction with 2 µM phenylephrine resulted in a significantly (*p* < 0.05) higher relative increase in the viscous modulus in the AIA, indicating a higher viscous response to VSMC contraction (Figure 7J).

### 3.9 The viscous properties of aortic tissue are modulated by the focal adhesion-F-actin interface

Finally, we aimed to investigate the role of focal adhesion activity and F-actin cytoskeleton integrity in the modulation of aortic viscoelasticity by pulse frequency. Therefore, we inhibited Src tyrosine kinase activity with PP2 to investigate the former, while F-actin was depolymerized with cytochalasin D. Interestingly, the decrease in the viscous modulus due to an increase in pulse frequency from 1 Hz to 25 Hz (denoted as “η_f_”) was attenuated by either 10 µM PP2 or 10 µM cytochalasin D (Figures 8A, B). Moreover, cytochalasin D had a significantly stronger effect on the ascending aorta compared to the infrarenal aorta, as indicated by a larger attenuation of η_f_ (Figure 8C). Src inhibition with PP2 had a similar relative effect on η_f_ attenuation in both types of aortic tissues.

## 4 Discussion

The present study aimed to unveil the complicated relationship between cyclic stretch, aortic viscoelasticity and the involvement of cellular adhesion (i.e., focal adhesion). Using an adapted Kelvin-Voigt model, we measured the *ex vivo* viscous and elastic properties of aortic tissue. We demonstrated that both the viscous and elastic moduli are increased in the abdominal infrarenal aorta compared to the thoracic aorta. Furthermore, the viscoelastic properties are similar in both the thoracic ascending and thoracic descending aorta. This indicates that the viscoelastic properties increase towards the periphery, which is in line with the findings of a previous study performed on aortic segments originating from sheep (Bia et al., 2005b). Whereas the elastic properties modulate the amplitude of pulsatile oscillations, the viscous properties attenuate high frequency pulsations and thereby counterbalance possible instabilities (Pontrelli and Rossoni, 2003). We conclude that the smaller elastic and viscous modulus in the thoracic ascending and thoracic descending aorta are necessary characteristics for optimal buffering of the ejected volume from the left ventricle. Indeed, a lower elastic modulus allows for higher distension of the aorta resulting in an increased compliance, allowing for optimal cushioning of blood flow (i.e., Windkessel effect) (Westerhof et al., 2009).

We have demonstrated that vascular smooth muscle cell contraction (VSMC) increases both the viscous and elastic modulus of the aorta. This is in concordance with other studies where VSMC contraction has been shown to increase pressure-radius hysteresis (i.e., artery wall viscosity) in human, rabbit and rat arteries (Greenwald et al., 1982; Boutouyrie et al., 1998; Armentano et al., 2006). Similarly, thoracic aortic wall viscosity in hypertensive patients decreased after VSMC relaxation with diltiazem, an L-type Ca2+ channel inhibitor, highlighting the role of VSMC tone in modulating arterial viscoelasticity (Stefanadis et al., 1997; Fransen et al., 2015). In the present study, VSMC contraction was associated with an increase in elastin fiber tortuosity and VSMC nucleus circularity. The latter could reflect a change in VSMC geometry upon contraction. Indeed, phenylephrine-induced contraction activated the “elastin-contractile unit” which led to the reorganization of elastin fibers to allow for vasoconstriction (Davis, 1993; Karimi and Milewicz, 2016). We further demonstrated that phenylephrine activates the focal adhesion (FA) complex under cyclic stretch, as shown by an increase in phosphorylation of focal adhesion kinase (FAK) after VSMC contraction. We hypothesize that activation of the FA complex led to the transmission of intracellular force, generated by actomyosin contraction, to the ECM which then resulted in elastin fiber reorganization. Consequently, we speculate that the increase in both the viscous and elastic modulus of the aorta after phenylephrine administration is a result of a reorganization of elastin fibers by contracting VSMCs.

Generally, it has been shown that cellular stiffness decreases when subjected to high amplitude cyclic stretch (Lai et al., 2022). Acute static and dynamic stretch have also been described to result in cytoskeletal fluidization and F-actin depolymerization in fibroblasts and smooth muscle cells, respectively, leading to decreased cellular stiffness (Lan et al., 2018; Walker et al., 2020). In the present study, a high, acute, pulsatile bout (i.e., 170 mmHg systolic pressure for 4 min) decreased the VSMC contraction-induced changes in viscoelastic properties of the thoracic descending aorta. Furthermore, VSMC contraction-induced elastin tortuosity was decreased after the acute high pulsatile bout as well. These results add to one of our previous studies where we have demonstrated that high pulsatile load decrease contraction-induced stiffening (Neutel et al., 2021). If VSMC contraction-induced elastin tortuosity is facilitated by cell-elastin connections, then the high pulsatile bout would have affected either such interactions and/or impede actomyosin contraction resulting in the observed straightening of the elastin fibers (Karimi and Milewicz, 2016). Indeed, increased cyclic stretch has been shown to decrease both actomyosin force generation and FA stability (Kaunas et al., 2011; Chen et al., 2012; De, 2018). Here, we have shown that Src-mediated phosphorylation of FAK at Tyr397, which is related to integrin engagement, is increased after phenylephrine administration and affected by pulsatility. Interestingly, whereas increased pulse pressure did not affect FAK phosphorylation, altered pulse frequency diminished PE-induced Tyr397 phosphorylation. These results are in disagreement with another study conducted in aortic segments from ferrets, where no increase in FAK phosphorylation (at Tyr397) was observed after phenylephrine administration (Min et al., 2012). However, their aortic segments were kept under static conditions and not under continuous cyclic loading such as in the present study. Our results suggest that at low frequency cyclic loading, which closely mimics the static environment as reported earlier mentioned study (Min et al., 2012), phenylephrine was unable to induce FAK phosphorylation (at Tyr397) and could explain these dissonant results. Nevertheless, we have demonstrated that a transient increase in pulsatility mitigates phenylephrine-increased viscoelastic properties of the aorta and that FAK activation, an important protein in the FA complex, is sensitive to pulsatility. However, it is peculiar that both a lower- and a higher-than normal frequency result in a decrease in FAK phosphorylation. One would expect a more linear behavior (e.g., FAK phosphorylation decreases with increasing frequency). Nonetheless, altering frequency clearly attenuated PE-induced FAK phosphorylation and therefore we believe this effect to be frequency-related. However, the mechanism leading to the decrease in FAK phosphorylation after changing stretch frequency is unclear and warrants further investigation to gain a better understanding of how FAK responds to mechanical signals such as stretch frequency. Yet, changes in phosphorylation of FAK do not fully explain how altered pulsatility affects viscoelasticity. Therefore, changes in the activity of other FA proteins such as paxillin and talin should be investigated in future studies. Alternatively, whereas the timescale of changes in signaling (such as FA phosphorylation) is in minutes, changes in potassium and calcium channel activity, which in turn modulate FA turnover, are potentially quicker and should be explored as well (Birukov, 2009; D'Souza et al., 2020).

We further evaluated the effect of *ex vivo* alterations in pulsatility on aortic viscoelasticity in different aortic regions. Altering pulsatility under basal, uncontracted, conditions, (either through altered pulse pressure or altered pulse frequency) changed the viscoelastic properties of aortic tissue. Increases in either pulse pressure or pulse frequency resulted in a decrease of both the viscous modulus and Peterson’s modulus. However, whereas increased pulse pressure resulted in a decreased elastic modulus, the opposite was observed for increased pulse frequency. In contrast to these findings, other studies demonstrated an increase in viscous properties of VSMCs and human aortic segments with increasing pulse frequency (Zhu et al., 2018; Amabili et al., 2020). In addition, the viscous properties of porcine LAD arteries were found to be independent of stretch frequency (Burton et al., 2017). Differences in the methods used to measure viscoelasticity and differences between human/porcine and murine aortic tissue could explain these dissonant results. Indeed, whereas an adaptation to the Kelvin-Voigt model has been used to study viscoelasticity in the present study, other nonlinear viscoelastic models might reveal differential effects of pulse frequency on viscoelasticity and should be investigated into further extent (Zhang et al., 2021).

In the present study, altered cyclic stretching conditions reversed VSMC contraction-induced changes in biomechanical properties but also altered viscoelastic parameters in uncontracted, basal conditions. Increased pulse frequency decreased the viscous modulus of all aortic segments, but this effect was attenuated after PP2 or cytochalasin D administration, inhibiting Src kinase activity and actin polymerization respectively. These results are similar to other findings, where cytochalasin D treatment mitigated energy dissipation under cyclic stretch in fibroblast microtissues (Walker et al., 2020). Therefore, we conclude that modulation of FA activity and/or F-actin fiber (re-)organization is responsible for the changes in viscoelasticity caused by altered cyclic stretch.

Whereas contraction and pulsatility affected the viscoelastic properties of the thoracic descending aorta, we have demonstrated that these effects are more pronounced in the abdominal infrarenal aorta. For example, phenylephrine contraction resulted in a higher increase in the viscous modulus in the abdominal infrarenal aorta compared to the thoracic descending aorta. Furthermore, the relationship between strain rate and the viscous modulus was stronger in the abdominal infrarenal aorta. The elastin fibers in the medial layer of the abdominal infrarenal aorta were found to be more tortuous than those in the medial layer of the thoracic descending aorta. Since we speculated that elastin fiber tortuosity is a result of intracellular force transmission through cellular adhesion to the matrix, we investigated FA activity in the abdominal infrarenal aorta. As expected, the abdominal infrarenal aorta was shown to have a different composition of focal adhesion proteins than the thoracic descending aorta. Whereas the thoracic descending aorta had more paxillin than the abdominal infrarenal aorta, talin was more abundant in the abdominal infrarenal aorta. In addition, we have demonstrated that, under physiological conditions, there is a higher interaction between vinculin and talin in the abdominal infrarenal aorta compared to the thoracic descending aorta. Talin and vinculin are considered to be part of the ‘structural module’ of focal adhesion proteins, whereas paxillin was described to be part of the ‘signaling module’ (Stutchbury et al., 2017). FA proteins from the structural module link the actin cytoskeleton to the ECM and are important for mechanosignaling (Atherton et al., 2016). This, taken together with our data, indicates that the thoracic descending aorta has a stronger “signaling module” whereas the abdominal infrarenal aorta has a more reinforced “structural” module. Furthermore, we have demonstrated that there is an increased amount of active talin-vinculin bonds in the AIA, an indication for more stabilized FA complexes. Interestingly, the stability of the structural FA proteins is influenced by extracellular matrix stiffness, as it has been shown that their half-life increased with substrate stiffness (Stutchbury et al., 2017). Therefore, more stable FA complexes in the abdominal infrarenal aorta could be a result of an increased ECM stiffness compared to the thoracic descending aorta. A more pronounced ‘structural FA module’ and higher FA stability in the abdominal infrarenal aorta explain its more pronounced viscoelastic response during VSMC contraction and altered cyclic stretch.

In conclusion, we have demonstrated that VSMCs contraction increases aortic viscoelasticity through increased focal adhesion activity and re-organization of elastin fibers. Increased cyclic stretch, such as during physical exercise and hypertension, may attenuate such contraction-induced changes by affecting focal adhesion function. Inversely, higher maturation or stability of the focal adhesion complex, as seen as in the abdominal infrarenal aorta, results in an enhanced viscoelastic response to pulsatility and VSMC contraction. Our findings show that the focal adhesion complex is an important factor in modulating aortic viscoelasticity and warrants further investigation.

## 5 Limitations

Whereas this study provides a detailed description of arterial viscoelasticity along the aortic tree, there are a few limitations that need to be taken into account. First, when referring to “pressure” throughout the manuscript, it needs to be kept in mind that this is in fact a calculated pressure, derived through the Laplace relationship. At any given preload (generated by the force-length transducer) the diameter of the aortic segments can be derived and therefore a “transmural pressure” can be estimated. Second, whereas visualization of elastin fiber tortuosity by histology yielded interesting results, it is not fully certain how this reflects *in vivo* tortuosity. Furthermore, a possible effect of formalin fixation on elastin fiber tortuosity cannot be fully excluded. A third limitation is associated with the experiments on studying the role of the focal adhesion-F-actin interface in modulating aortic viscoelasticity. Here, PP2 was administered first, followed by multiple cycles of washing, after which the aortic segments were incubated with cytochalasin D. This order is important, since PP2 is reversible whereas cytochalasin D results in irreversible damage to the vascular cells. Therefore, possible cross-over effects of PP2 could not be studied. Furthermore, it is known that PP2 is not selective for Src kinases, and therefore other possible effects can occur when incubating aortic segments with PP2. Lastly, only male mice were used in this study as this study was designed to study the fundamental effects of physical parameters (e.g., stretch frequency) and biological mechanisms (e.g., focal adhesion composition) on biomechanical properties of individual aortic segments as well as regional heterogeneity in the same animal. Nonetheless, future studies should include sex/gender as a study parameter to gain a better understanding of aortic viscoelasticity.

## Data Availability

The original contributions presented in the study are included in the article/Supplementary Material, further inquiries can be directed to the corresponding author.
