# Supplementary material for: The effect of cyclic stretch on aortic viscoelasticity and the putative role of smooth muscle focal adhesion
Source: Front Physiol. 2023 Aug 11;14:1218924. doi: 10.3389/fphys.2023.1218924 (PMC10450742; doi:10.3389/fphys.2023.1218924)
Supplement: Supplementary file 1 [file DataSheet1.docx]

Supplementary Material

The Effect of Cyclic Stretch on Aortic Viscoelasticity And The Putative Role Of Smooth Muscle Focal Adhesion

**Cédric H.G. Neutel^1*^, Callan D. Wesley^1^, Guido R.Y. De Meyer^1^, Wim Martinet^1^, Pieter-Jan Guns^1^**

^1^Laboratory of Physiopharmacology, University of Antwerp, Campus Drie Eiken, Antwerp, Belgium

*** Correspondence:** Cédric H.G. Neutel: cedric.neutel@uantwerpen.be

# Supplementary Data

## Supplementary Figure 1


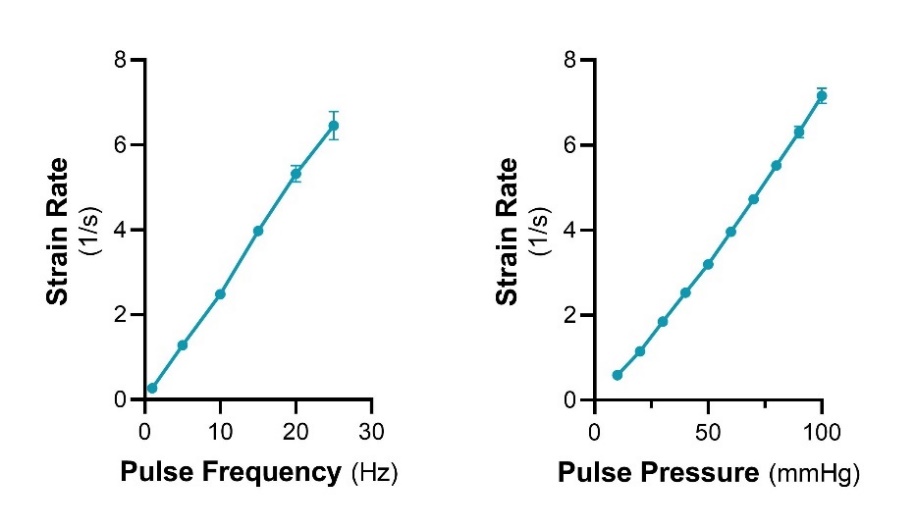


**Supplementary Figure 1.** **Ex vivo increases in either pulse pressure or pulse frequency result in increased strain rate**. By increasing either pulse pressure or pulse frequency ex vivo, an increase in strain rate is observed, calculated as the amount of strain per cycle.

## Supplementary Figure 2


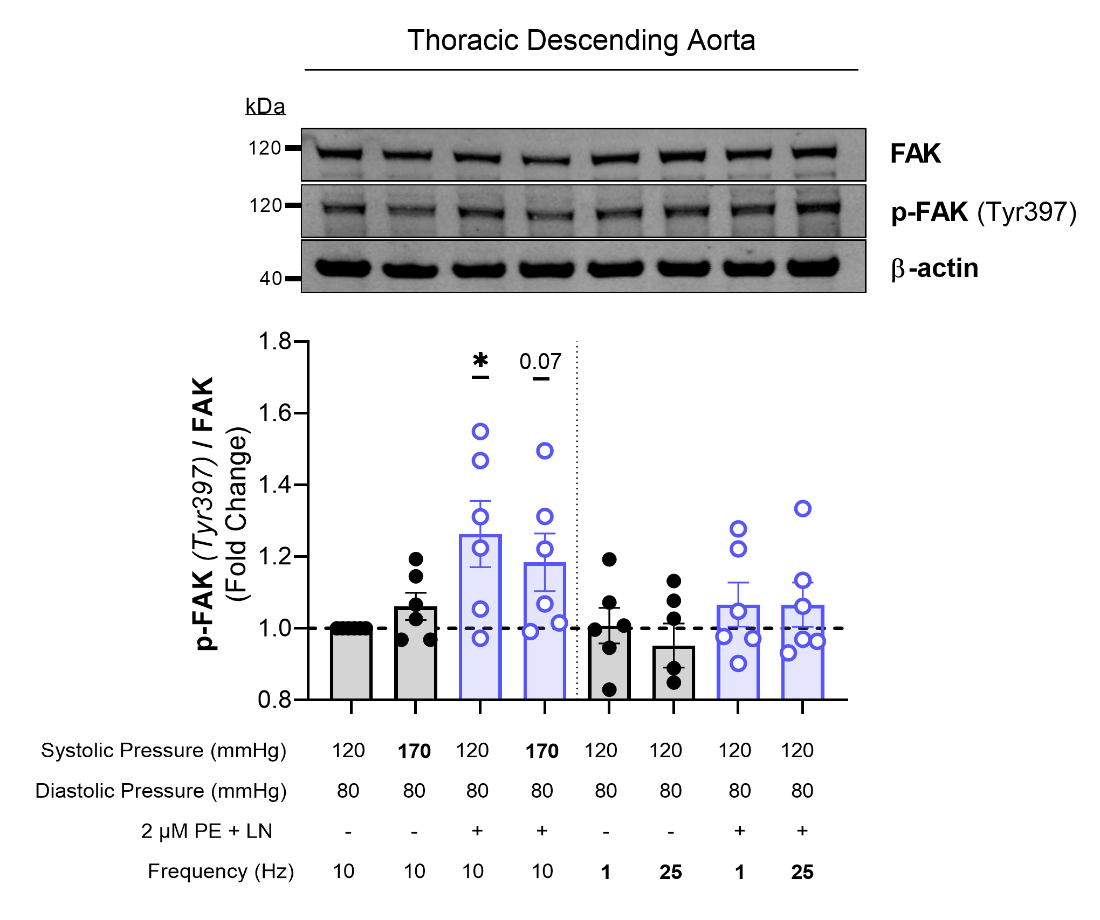


**Supplementary Figure 2.** **Phenylephrine-induced FAK phosphorylation is dependent on pulsatility** .Thoracic aortic segments were either pre-contracted with 2 µM phenylephrine (+ 300 µM L-NAME) or kept under basal conditions while oscillating between 80 and 120 mmHg diastolic and systolic pressure, respectively. After 30 minutes, aortic segments were subjected to either high/normal pulsatility (systolic pressure of either 120 or 170 mmHg) or high/low pulse frequency (1 Hz or 25 Hz). After 4 minutes, the conditions were returned to normal pulsatility and frequency (80-120 mmHg, 10 Hz). One minute after the acute pulsatility/frequency bout, the segments were collected in Laemmli buffer and a series of immunoblots were performed for phospho-FAK (Tyr397) and FAK. Phenylephrine induced a significant (p<0.05) increase in p-FAK when oscillating at 80-120 mmHg (10 Hz). After the acute pulsatility bout (170 mmHg systolic pressure, 4 minutes), p-FAK was still increased (non-significant difference vs control after statistical analysis (p=0.07)). Interestingly, phenylephrine-mediated increase in p-FAK was absent after both frequency bouts (1 Hz and 25 Hz). Statistical analysis was performed by using a One-sample T test on Log2 transformed FC (vs control) data, hypothetical value=0. n=6. *p<0.05. **(p-)FAK** = (phosphorylated-)Focal Adhesion Kinase; **PE** = Phenylephrine; **LN** = L-NAME

## Supplementary Figure 3


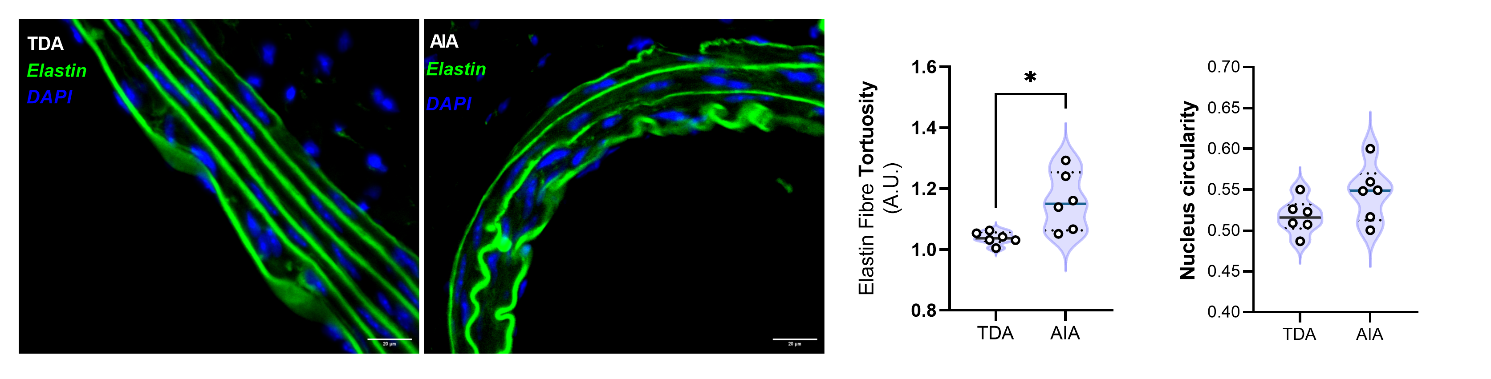


**Supplementary Figure 3. Elastin fibers are more tortuous in the abdominal infrarenal aorta under physiological conditions**. Thoracic descending and abdominal infrarenal aortic segments were formaldehyde-fixed under high frequency (10 Hz) cyclic stretch at 80 and 120 mmHg diastolic and systolic pressure, respectively. The elastin fibers in the abdominal infrarenal aorta displayed a significantly higher level of tortuosity than the elastin fibers in the thoracic descending aorta (A). No differences in nucleus circularity of medial cells were observed between the aortic segments (B). Statistical analysis was performed using an unpaired T-test. n=6. *p<0.05. **TDA** = Thoracic Descending Aorta; **AIA**= Abdominal Infrarenal Aorta
